# CircRNA based bi-antigen vaccines against mpox virus induce potent and durable cross-protection in mice

**DOI:** 10.1186/s43556-026-00474-9

**Published:** 2026-05-28

**Authors:** Jinge Zhou, Chen Wang, Yan Wu, Baoxin Zhao, Yun Yang, Tianxi Ye, Kaiyue Zhang, Fangxu Li, Jiang Hu, Kai Zhang, Fang Liu, Weiqi Wang, Chunhua Wang, Zefeng Wang, Xia Chuai, Sandra Chiu

**Affiliations:** 1 State Key Laboratory of Virology and Biosafety, Center for Biosafety Mega Science, Wuhan Institute of Virology, Chinese Academy of Sciences, Wuhan, Hubei 430207 China; 2 University of Chinese Academy of Sciences, Beijing, 100049 China; 3 Research and Development Department, Shanghai CirCode Biomedicine Co.Ltd, Shanghai, 200131 China; 4 Division of Life Sciences and Medicine, University of Science and Technology of China, Hefei, Anhui 230027 China; 5 Key Laboratory of Anhui Province for Emerging and Reemerging Infectious Diseases, Hefei, Anhui 230027 China; 6 Department of Clinical Laboratory, Xiangyang Central Hospital, Affiliated Hospital of Hubei University of Arts and Science, Xiangyang, Hubei Province 441021 China; 7 Department of System Biology, School of Life Science, Southern University of Science and Technology, Shenzhen, China; 8 Department of Laboratory Medicine, Division of Life Sciences and Medicine, The First Affiliated Hospital of USTC, University of Science and Technology of China, Hefei, Anhui 230031 China

**Keywords:** Circular RNA vaccines, Mpox virus, Immune responses, Long-lasting protection, Mouse model

## Abstract

The ongoing epidemic of mpox highlights the urgency of developing novel and effective vaccines against mpox virus (MPXV). Circular RNA (circRNA) has emerged as a promising novel vaccine platform due to its high stability, resistance to exonuclease-mediated degradation, and potential to support prolonged antigen expression. Based on our lipid nanoparticle (LNP)-encapsulated circRNA vaccine platform, we developed two novel bi-antigen circRNA vaccines cirEV and cirMV, which respectively encode the combination of MPXV extracellular enveloped virion (EEV) antigens (A35R and B6R) or intracellular mature virion (IMV) antigens (A29L and M1R). The two MPXV circRNA vaccines alone or mixed as a combination vaccine successfully induce high levels of antigen-specific antibody responses and cellular immune responses against the MPXV antigens and protect mice from lethal vaccinia virus (VACV) challenge in a dose-dependent manner. Furthermore, both circRNA vaccines can provide complete long-term cross-protection against lethal VACV challenge at day 260 after first immunization, indicating the durability of vaccine-induced protective immunity. Notably, vaccine-induced cellular immune responses play a crucial role in immune protection and virus clearance in mice. Our study provides critical insights to understand the protective mechanisms underlying circRNA vaccines in an *orthopoxvirus* surrogate model, demonstrating that bi-antigen circRNA vaccines represent a promising multivalent vaccine platform.

**Supplementary Information:**

The online version contains supplementary material available at 10.1186/s43556-026-00474-9.

## Introduction

Mpox (previously known as monkeypox) is caused by mpox virus (MPXV) which belongs to the *Orthopoxvirus* genus of *Poxviridae* family. MPXV has two distinct clades: the Congo Basin (Central African) clade (clade I) and West African clade (clade II). Clade I was reported to cause greater severity of disease and death, while Clade II has a lower virulence [1]. Historically, mpox has been endemic predominantly across Central and West Africa. However, a sudden outbreak of mpox caused by MPXV clade IIb began in May, 2022 and rapidly spread worldwide [2]. And in September 2023, another outbreak of mpox began in the Democratic Republic of the Congo (DRC) caused by the more virulent MPXV clade Ib strains. It quickly spread to nearby areas and even outside of Africa in Stockholm, Sweden, and Thailand [3, 4]. These outbreaks remain ongoing and the WHO declared it a PHEIC in August 2024. Thus, it highlights the urgent need to develop effective prevention strategies and treatments against mpox to reduce the disease burden during outbreaks.

As a member of the *Orthopoxvirus*, MPXV shares high genetic homology with variola virus (VARV) and vaccinia virus (VACV), and smallpox vaccines have been experimentally demonstrated to confer highly cross-protection against MPXV. Currently, three smallpox vaccines including ACAM2000, JYNNEOS and LC16m8 have been authorized to prevent mpox [5, 6]. ACAM2000 is a second-generation live attenuated vaccine, which was showed to provide effective protection against mpox when applied after exposure. However, ACAM2000 is associated with significant adverse effects, including dermatitis, myocarditis and pericarditis [7]. JYNNEOS is a non-replicating vaccine derived from modified vaccinia Ankara (MVA), which is safer for use in immunocompromised populations than ACAM2000. However, JYNNEOS vaccination provided weaker immune protection in individuals with poor immune than that in healthy people, so the efficacy and durability of JYNNEOS remain to be further investigation and monitored [8]. LC16m8 is an attenuated live vaccine derived from the Lister strain, which was first created and licensed in Japan [9], and on 19 November 2024, WHO granted Emergency Use Listing (EUL) for the LC16m8 mpox vaccine [10]. Although the preclinical studies in monkey demonstrated that LC16m8 could induce the neutralizing antibodies and cell-mediated immune responses, effectively preventing viremia and the onset of monkeypox-related lesions after MPXV infection, the efficacy in mpox in real-world remains to be monitored. The above smallpox vaccines are all recommended for preexposure prophylaxis for high-risk populations (pre-exposure) and those exposed to the virus (post-exposure) [11], however the efficacy may decline over time [12]. Thus, a safe and efficient MPXV specific vaccine is still urgently required.

mRNA-based vaccines have been developed to prevent MPXV infection due to their advantages such as quick development and evaluation, high efficacy, strong safety as well as low-cost manufacture [13]. Vaccination with these mRNA-based vaccines encoding MPXV antigens can induce both strong humoral and cellular immune responses and demonstrated protective efficacy against viral challenge in animal models [14]. However, the conventional mRNA vaccines face inherent limitations. For example, they are highly susceptible to degradation and often with high immunogenicity [14, 15]. CircRNAs are a class of single-stranded, covalently closed RNA molecules that exhibit greater resistance to exonuclease-mediated degradation. Compared to linear mRNAs, circRNAs demonstrate enhanced structural stability and sustain prolonged protein expression [16, 17]. Thus circRNA-based vaccine is becoming a promising platform in the prevention of infectious diseases and the treatment of tumors [18]. We previously developed four monovalent circRNA-based vaccines respectively encoding MPXV antigens (A29L, A35R, B6R, and M1R) and evaluated their efficacy against lethal VACV challenge in a murine model. Accumulated evidence indicates that both MPXV extracellular enveloped virion (EEV) proteins (A35R and B6R) and intracellular mature virion (IMV) proteins (M1R and A29L) serve as protective antigens. These proteins have been shown to elicit neutralizing antibodies in animals and humans [19–21]. Mice immunized with either individual circRNAs (cirA29L, ciA35R, cirB6R, and cirM1R) or the combination of the four vaccines (cirMix4) induced high levels of MPXV antigen-specific humoral and cellular immune responses. Strikingly, cirMix4 significantly protected mice against lethal VACV challenge, demonstrating the potential of circRNA vaccines platforms for developing next-generation MPXV and even *orthopoxvirus* vaccines [22]. However, the combination of these individual circRNA vaccines will increase production costs, and thus limiting their broad application. Furthermore, the durability of immune protection still requires further evaluation. Therefore, the combination of multiple protective antigens from both EEV or IMV of MPXV will be an ideal choice in the development of novel MPXV vaccines [23].

In this study, by using our proprietary circRNA vaccine technology platform [22], we constructed two circRNA vaccines: cirEV expressing MPXV EEV proteins A35R-B6R and cirMV expressing IMV proteins A29L-M1R. Both vaccines were designed to contain two tandem antigens in a single circRNA and delivered by lipid nanoparticles (LNPs). We evaluated the immunogenicity and protective efficacy of the two circRNA vaccines alone or in combination in mouse model by assessing the long-term immunity and protection against VACV challenge. Our results demonstrate that the two-component circRNA vaccines (cirEV and cirMV) and a four-antigen circRNA vaccine (cirE&M Mix) successfully induce high levels of antigen-specific antibody and cellular immune responses against the MPXV antigen and protect mice from VACV challenge in a dose-dependent manner. Furthermore, both the two-component MPXV circRNA vaccines alone or in combination can induce durable antigen-specific antibody responses and provide complete cross-protection against lethal VACV challenge at the 260 days after first immunization. Our data demonstrate that the bi-antigen circRNA vaccines could induce potent cellular immunity which may be mainly responsible for the long-term protection against virus challenge, providing a promising multivalent vaccine platform.

## Results

### Characterization of bi-antigen MPXV circRNA vaccines

**Fig. 1:**
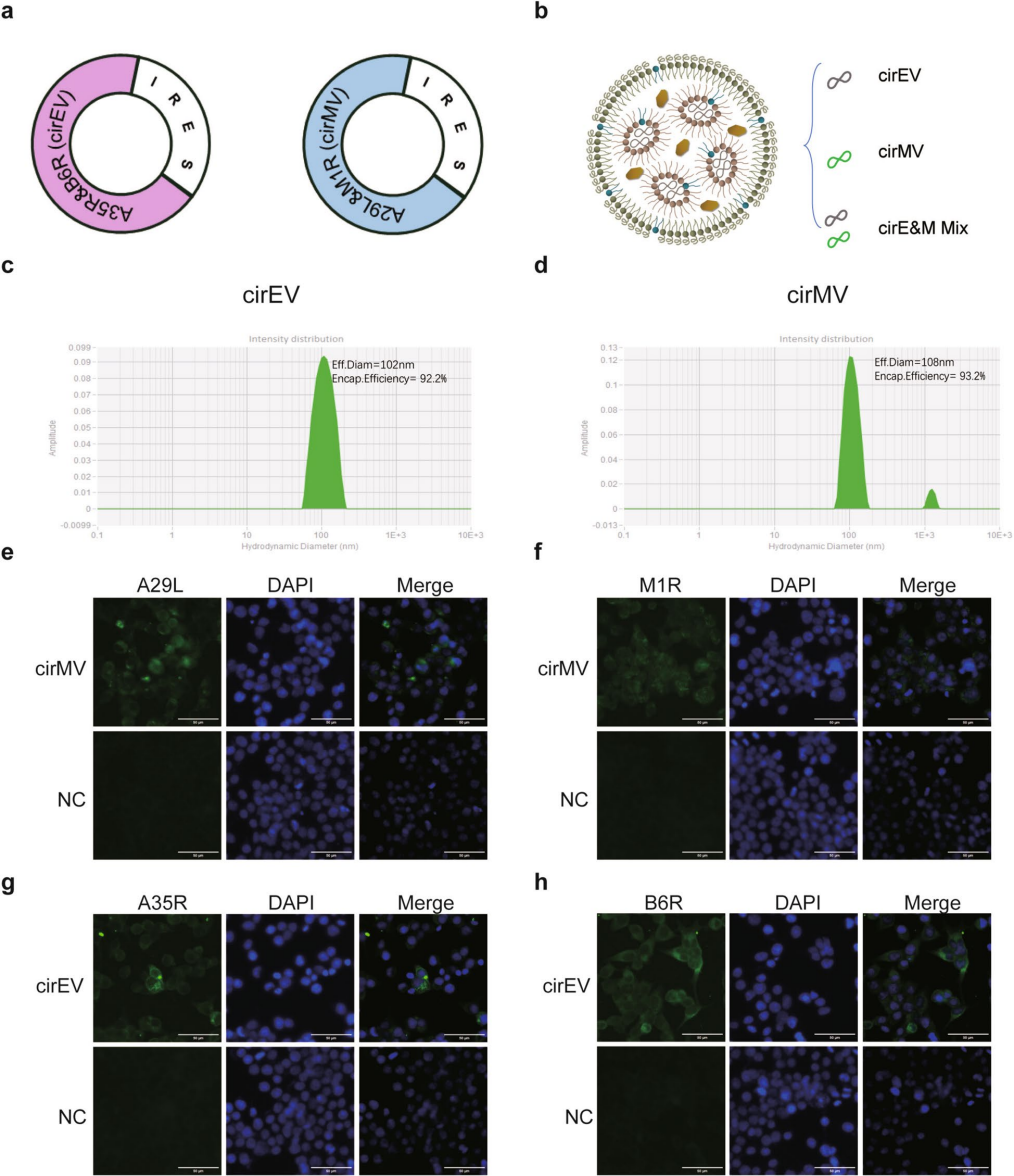
Design and characterization of MPXV circRNA vaccines. **a**-**b** Design of circRNA. The circular RNAs constructed express the MPXV A35R-B6R and A29L-MIR antigens. **c**-**d** The particle diameter and encapsulation efficiency of each circRNA construct. **e**–**h** The expression of the MPXV-specific antigens A29L, M1R, A35R, and B6R in HEK293T cells was detected by Immunofluorescence assays (IFAs), Scale bar = 50 μm

This study aims to develop circRNA vaccines targeting both the EEV and IMV proteins of MPXV. Specifically, we selected the EEV antigens B6R and A35R, along with the IMV antigens A29L and M1R, to construct MPXV circRNA vaccines encoding two tandem antigens. These vaccines were designated as cirEV (targeting EEV) and cirMV (targeting IMV) respectively. CircRNAs featuring self-splicing introns from group II were designed to ensure efficient protein translation (Fig. 1a). The co-circularization efficiencies of the circRNAs are as follows: cirMV at 46.2% and cirEV at 51.5%. The circRNA vaccines, cirEV and cirMV, were encapsulated in lipid nanoparticles (LNPs) to generate two multi-antigen vaccine formulations (Fig. 1b). The encapsulation efficiency for cirEV was 92.2% and 93.2% for cirMV, with both LNP formulations exhibiting an average diameter of approximately 100 nm (Fig. 1c-d). Following transfection of cirEV and cirMV into HEK293T cells, immunofluorescence analysis and western blotting demonstrated successful expression of all four target proteins (B6R, A35R, A29L, and M1R) (Fig. 1e-h, Fig.S1).

### Bi-antigen MPXV circRNA vaccines induce antigen-specific antibody responses in mice

**Fig. 2:**
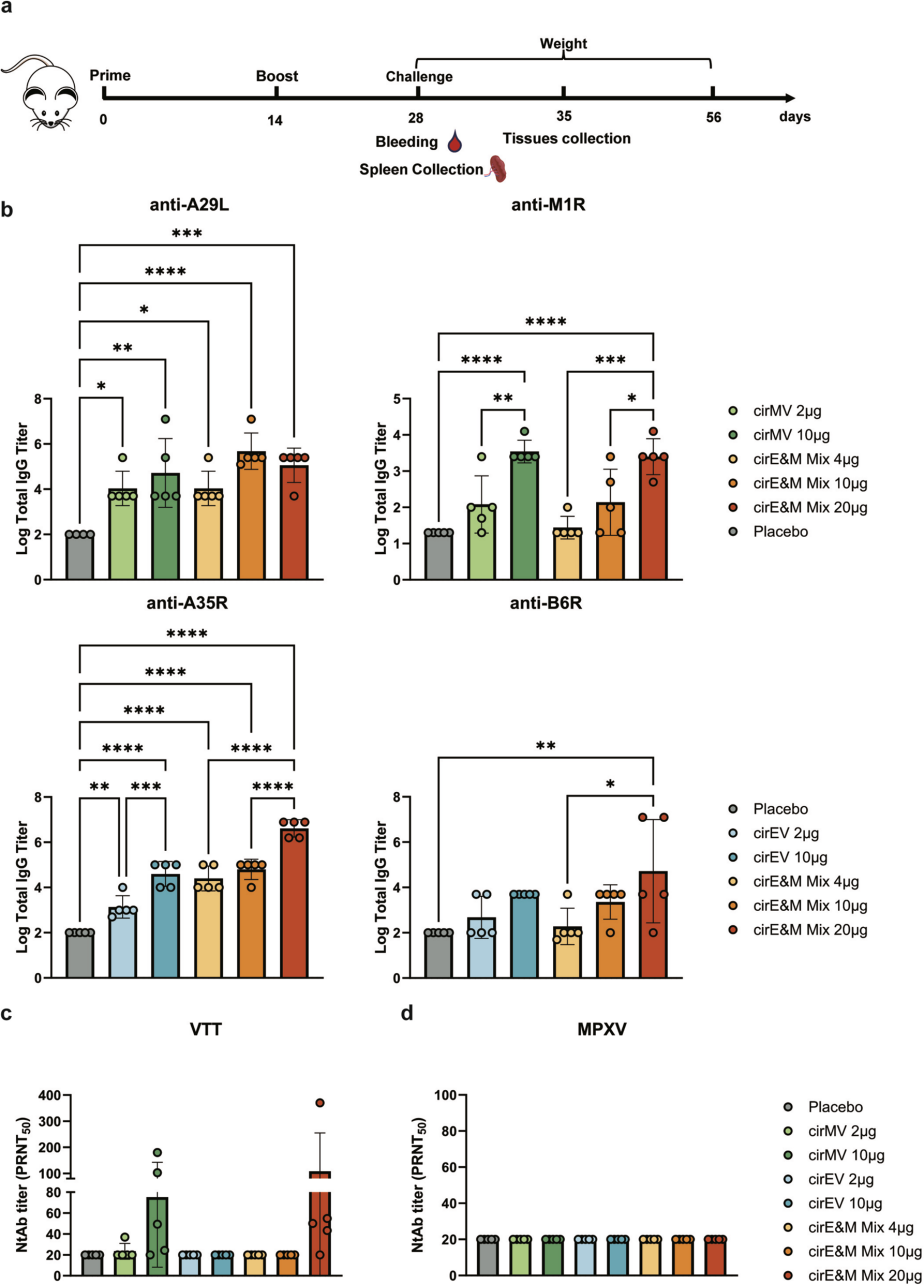
Humoral immune responses in mice after vaccination with Bi-antigen MPXV circRNA vaccines. **a** Immunization and challenge schematic diagram. Female 6-week-old BALB/c mice were randomly divided into 8 groups (n = 11) and were immunized twice with MPXV circRNA vaccines or placebo at 2-week intervals. Samples were obtained at 28 days following the initial immunization for antibody detection. Six mice from each group were intranasally challenged 1 × 10^6^ PFU of VTT on day 28 after the first immunization. **b** MPXV A29L, M1R, A35R and B6R specific antibody titers were assessed using ELISA (n = 5). **c** The titers of cross-neutralizing antibodies against VTT were measured using a PRNT assay (n = 5). **d** The titers of neutralizing antibodies against MPXV were measured using a PRNT assay (n = 5). Data are presented as the mean ± SEM. Significance was calculated using one-way ANOVA with multiple comparison tests (*p < 0.05, **p < 0.01, ***p < 0.001, **** *p* < 0.0001)

A prime-boost immunization regimen was administered to BALB/c mice using the two bi-antigen vaccines (cirEV & cirMV) and a mixture of cirEV and cirMV (cirE&M Mix) (Fig. 2a). Following two immunizations, all the vaccines elicited MPXV antigen-specific antibody responses against all targeted components. The antibody levels exhibited a dose-dependent increase following vaccination. The quadrivalent vaccine cirE&M Mix generated antibodies against all four antigens, though with comparatively weaker responses to M1R. These results suggest that circRNA vaccines can effectively induce MPXV antigen-specific antibody responses in mice (Fig. 2b). This may be related to differences in antigen expression of one vaccine construct.

circRNA vaccines-induced neutralizing antibody were measured using the PRNT50 assay against both VTT and MPXV in immunized mouse sera. As shown in Fig. 2c, neutralizing antibodies against VTT were only detected in the 10 μg cirMV group and 20 μg cirE&M Mix group. However, the neutralizing antibody titers against MPXV were below the detection threshold for all vaccinated groups (PRNT_50_ < 20) (Fig. 2d). As previously observed [22], M1R was the primary antigen for inducing neutralizing antibodies. However, due to the low binding antibody titers for M1R in earlier assays, the overall neutralizing antibody levels remained relatively low. In summary, while all circRNA vaccine formulations (cirEV/cirMV and quadrivalent cirE&M Mix) elicited dose-dependent MPXV antigen-binding antibodies, their neutralizing activity remained limited.

### Bi-antigen MPXV circRNA vaccines elicit strong cellular immune responses in mice

**Fig. 3:**
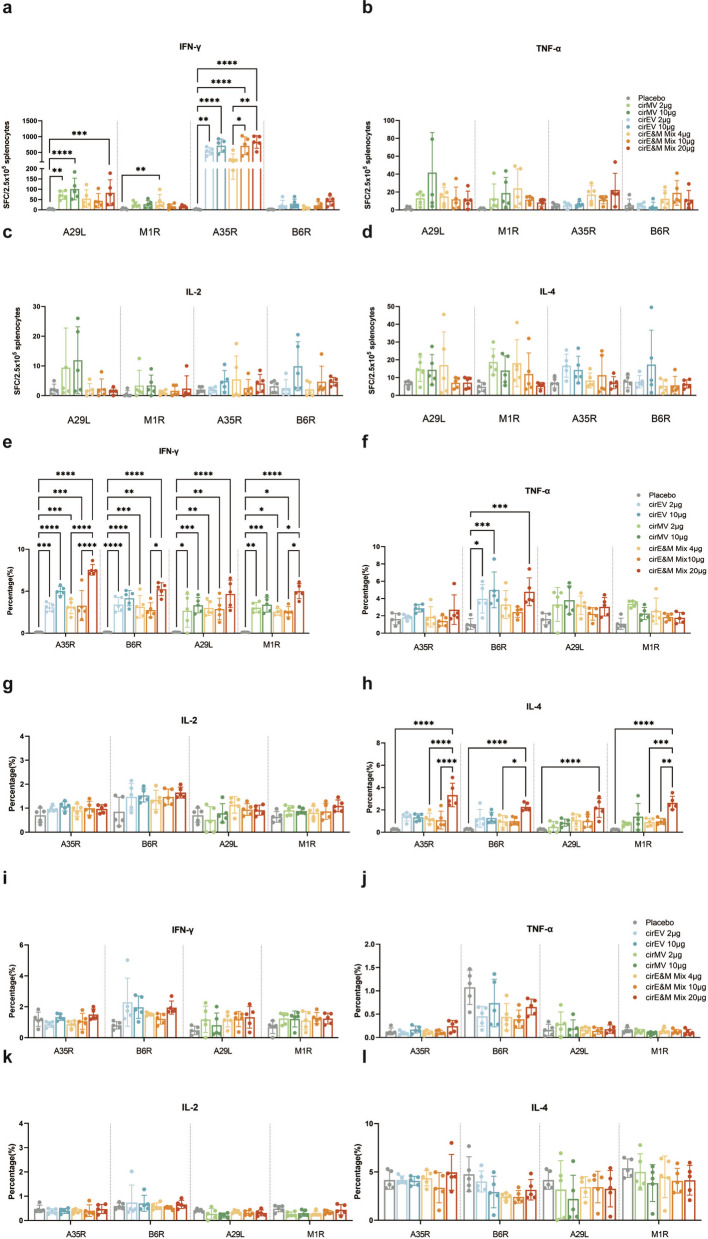
Cellular immune responses in mice after vaccination with Bi-antigen MPXV circRNA vaccines. **a**-**d** Splenocytes were isolated 2 weeks after the second immunization (n = 5), and ELISPOT assay was used to detect the numbers of T cells producing IFN-γ-, TNF-α-, IL-2-, and IL-4 stimulated with MPXV-A29L, A35R, M1R, and B6R peptides pools. **e**–**h** MPXV antigens specific IFN-γ, TNF-α, IL-2 and IL-4 in CD4^+^ T cells were detected by flow cytometry (n = 5). **i**-**l** MPXV antigens specific IFN-γ, TNF-α, IL-2 and IL-4 in CD8^+^ T cells were detected by flow cytometry (n = 5). The data are presented as the mean ± SEM. Significance was calculated using one-way ANOVA with multiple comparison tests (*p < 0.05, **p < 0.01, ***p < 0.001, **** *p* < 0.0001)

In addition to antibody-mediated immunity, cell-mediated immune responses are also crucial in preventing viral infections. Thus, MPXV antigen specific T cell immune responses were first evaluated using ELISpot assay. Compared to the placebo group, the cirEV vaccine group significantly increased the number of A35R specific IFN-γ-secreting cells. In contrast, B6R peptides stimulation elicited weaker cellular responses (Fig. 3a). The cirMV vaccine formulation induced measurable cellular immunity to both IMV antigens (A29L and M1R), but the specific cellular immune responses were relatively low (Fig. 3a). In contrast, the quadrivalent vaccine cirE&M Mix induced a significant cellular immune response, with higher levels of IFN-γ-secreting cells (Fig. 3a). A dose-dependent enhancement of cellular immunity was also observed across all vaccines immunized groups. Whereas, cells secreting other cytokines were not significantly enhanced (Fig. 3b-d). The overall ELISpot response profile was further summarized in a heatmap, which confirmed that cirE&M Mix induced broader and stronger antigen-specific cellular immune responses than the single bi-antigen vaccine formulations (Fig. S2).

On day 28 post-immunization, MPXV antigen-specific T cell responses were further characterized by intracellular cytokine staining (ICS). The cirEV, cirMV and cirE&M Mix vaccines induced a stronger CD8^+^ T cell immune response, especially among CD8^+^ T cells producing IL-4 and IFN-γ (Fig. 3e-h). In contrast, no significant differences in the proportion of IFN-γ, IL-2, TNF-α, IL-4-secreting CD4^+^ T cells were observed across all circRNA vaccine groups (Fig. 3i-j), suggesting those vaccines may preferentially activate CD8^+^ T cells (cell-mediated immunity) while being less dependent on antigen-presenting cells, resulting in a weaker CD4^+^ T cell (helper T cell) response. The overall ICS profile was also visualized using heatmaps, which showed the cytokine-producing CD8^+^ and CD4^+^ T cell responses induced by different vaccine formulations following antigen stimulation (Fig. S3).

### Bi-antigen MPXV circRNA vaccines provide protection against VTT in mice

**Fig. 4:**
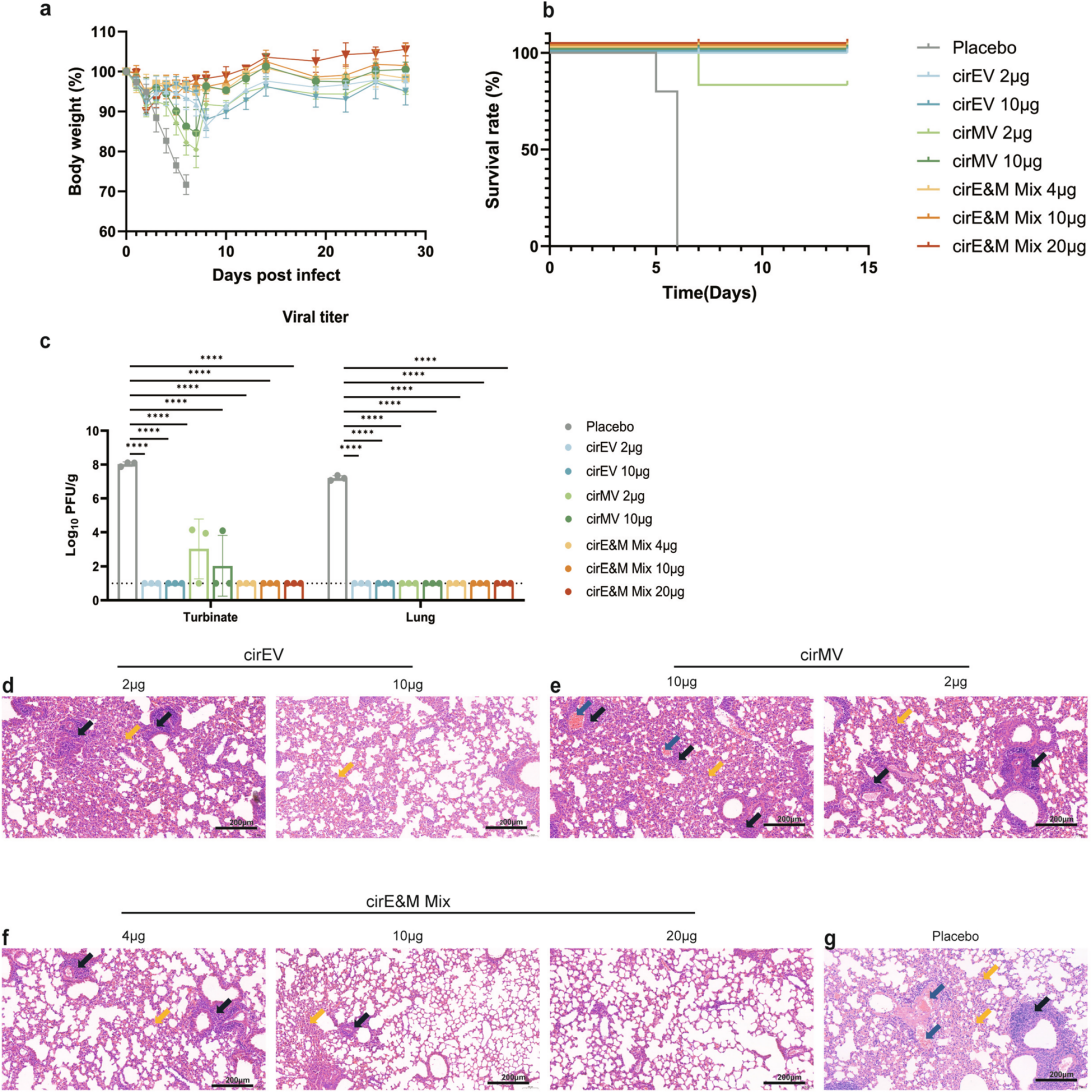
Protection by the bi-antigen MPXV circRNA vaccines against lethal VTT challenge in mice. On day 28 post-primary immunization, mice (n = 6) were intranasally challenged with 1 × 10^6^ of PFU VTT in 50 µl volume. **a**-**b** Weight loss and survival of mice following VTT challenge. **c** On day 7 post VTT challenge, lung tissues were collected and viral loads were determined by standard plaque assay in Vero E6 cells (n = 3). **d**-**g** H&E staining of lung sections from mice after VTT challenge. Scale bar = 200 μm. The blue arrow shows pulmonary vein congestion, the black arrow shows lymphocyte infiltration, and the yellow arrow shows alveolar congestion. The data are presented as the mean ± SEM. Significance was calculated using one-way ANOVA with multiple comparison tests (*p < 0.05, **p < 0.01, ***p < 0.001, **** *p* < 0.0001)

To assess the protective efficacy of each vaccine candidate, BALB/c mice (n = 6) were challenged intranasally with 1 × 10^6^ PFU of VACV Tian Tan (VTT) two weeks after the second immunization. Body weight changes post-challenge served as an indicator of overall health. VTT infection led to slight weight loss in all groups from the first day, with the placebo group showing continuous weight loss, eventually reaching euthanasia criteria (25% weight loss) or displaying severe symptoms (Fig. 4a). All mice in the placebo group succumbed to infection by day 6 post-infection, while one mouse in the 2 µg cirMV group died at the same timepoint (Fig. 4b). In contrast, the other vaccine groups regained weight after an initial loss. Consistent with our prior findings [22], the quadrivalent vaccine group cirE&M Mix exhibited the mildest weight changes and symptoms, with the fastest recovery to normal weight, with 100% survival throughout the study period (Fig. 4a-b).

We further quantified the viral loads in nasal turbinates and lung tissues using plaque assay at day 7 post-VTT challenge. Compared to placebo controls, all vaccinated groups showed significant reductions in infectious viral particles (Fig. 4c). A trace amount of residual virus was detected in the nasal turbinates of cirMV-vaccinated mice, which is the site of infection. The quadrivalent cirE&M Mix demonstrated superior protection, completely clearing virus from all tissues even at the lowest dose (2 μg of each component). Notably, viral suppression was more pronounced in lung tissue compared to the primary infection site in nasal turbinates (Fig. 4c).

To further assess the protective efficacy of the circRNA vaccines against VTT infection, we performed H&E staining of lung tissues to evaluate virus induced damage. As applicated in Fig. 4d-g, mice in placebo group exhibited severe necrosis in alveoli and bronchi, as judged by unclear structures, necrotic debris, and extensive inflammatory cell infiltration. Lymphocyte infiltration was also observed around blood vessels and bronchi (indicated by arrows in the figure), as well as pulmonary congestion, alveolar enlargement, and alveolar cavity expansion (Fig. 4g). For the cirEV group, significant inflammatory cell infiltration was still present at 2 µg dose, while the 10 μg dose demonstrated significant reduction of inflammation (Fig. 4d). In the cirMV group, both 2 µg and 10 µg doses showed significant inflammatory infiltration, thickened lung tissue, smaller alveoli, and some pulmonary congestion (Fig. 4e). The mixed bi-antigen vaccine cirE&M Mix immunization showed dose-dependent improvement in pulmonary histopathology: while the 2 μg dose group exhibited moderate inflammatory infiltration, the 10 μg dose achieved near-normal lung architecture with only focal residual inflammation. The 20 μg high-dose vaccination group of cirE&M Mix demonstrated optimal protection, exhibiting minimal inflammatory infiltration and near-complete preservation of pulmonary architecture (Fig. 4f).

### Bi-antigen MPXV circRNA vaccines provide long-term antibody responses and protection against VTT in mice

**Fig. 5:**
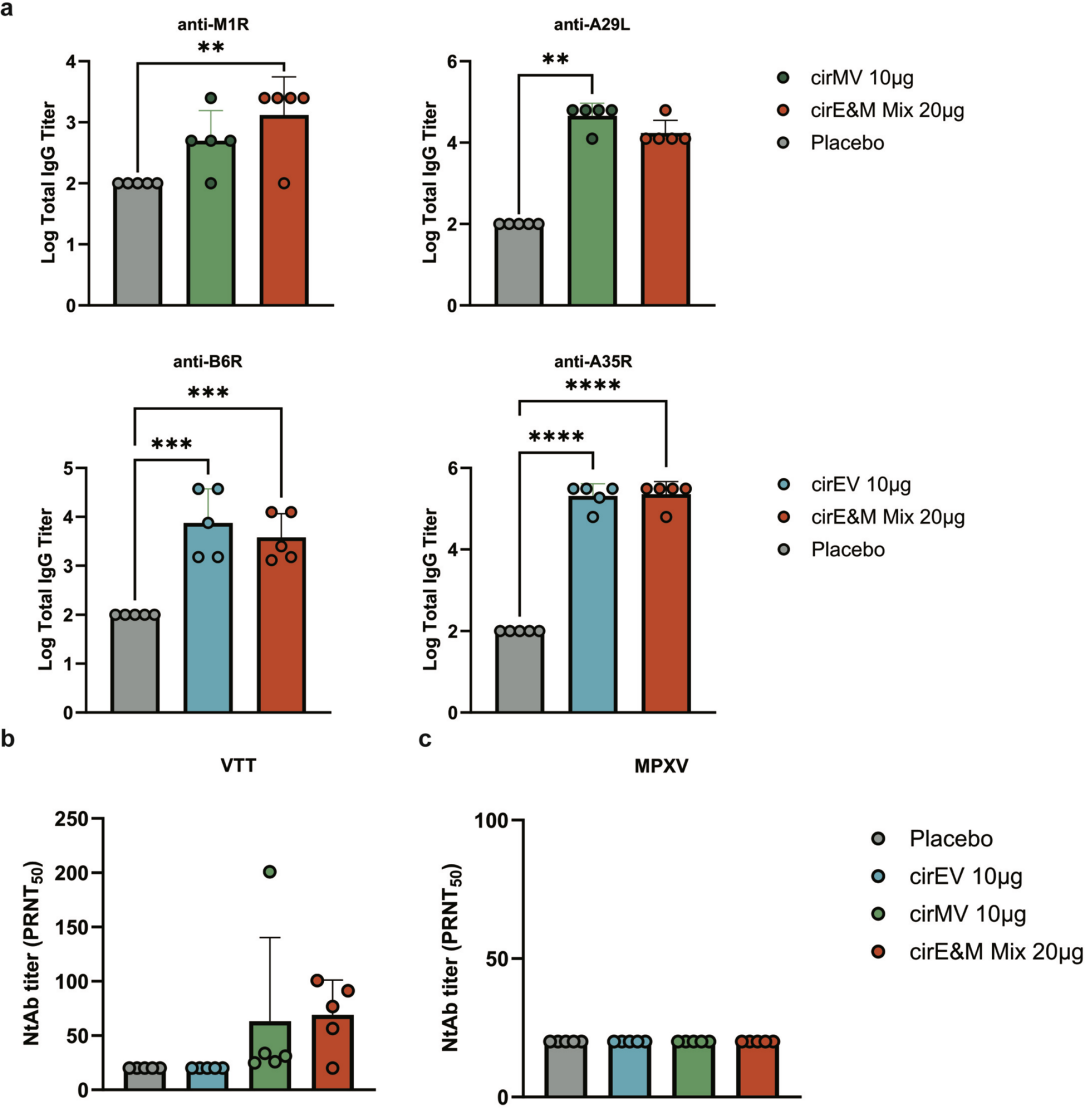
The long-term IgG and VACV- and MPXV-neutralizing antibody responses in mice immunized with bi-antigen MPXV circRNA vaccines. Group mice (n = 5) were immunized with either 10 μg cirMV and cirEV, 20 μg cirE&M Mix, or placebo on days 0 and 14. Serum samples were collected on day 260 post-primary immunization to assess the long-term MPXV-specific antibody responses, followed by a lethal VTT challenge to evaluate protective efficacy. **a** Serum IgG antibody titres of M1R, A29L, B6R and A35R were detected using ELISA until 260 days after the initial immunization. **b** The VTT-cross neutralizing antibody titres determined by the PRNT assay on day 260 post initial immunization. **c** Neutralizing antibody titres for MPXV on day 260 post initial immunization. Data are shown as the mean ± SEM. Significance was calculated using one-way ANOVA with multiple comparison tests. *p* values are indicated by *p < 0.05, **p < 0.01, ***p < 0.001 and ****p < 0.0001

To evaluate long-term immune responses, BALB/c mice (n = 10) were immunized twice with 10 μg bi-antigen MPXV circRNA vaccines (cirEV & cirMV) or 20 μg mixed bi-antigen vaccine cirE&M Mix, respectively. On day 260 post-immunization, serum samples were collected from all mice for assessment of humoral immune responses. As shown in Fig. 5a, high levels of MPXV B6R, A35R, A29L and M1R-specific binding antibodies were still detectable in all immunized mice at 260 days post-prime immunization. While low levels of neutralizing antibodies against VTT were detected in the cirMV and cirE&M Mix immunization groups, the MPXV-specific neutralizing antibodies were undetectable in all vaccinated mice at this time point (Fig. 5b-c). Notably, the neutralizing activity in cirMV and cirE&M sera showed comparable persistence to day 28 levels, with no significant decline observed.

**Fig. 6:**
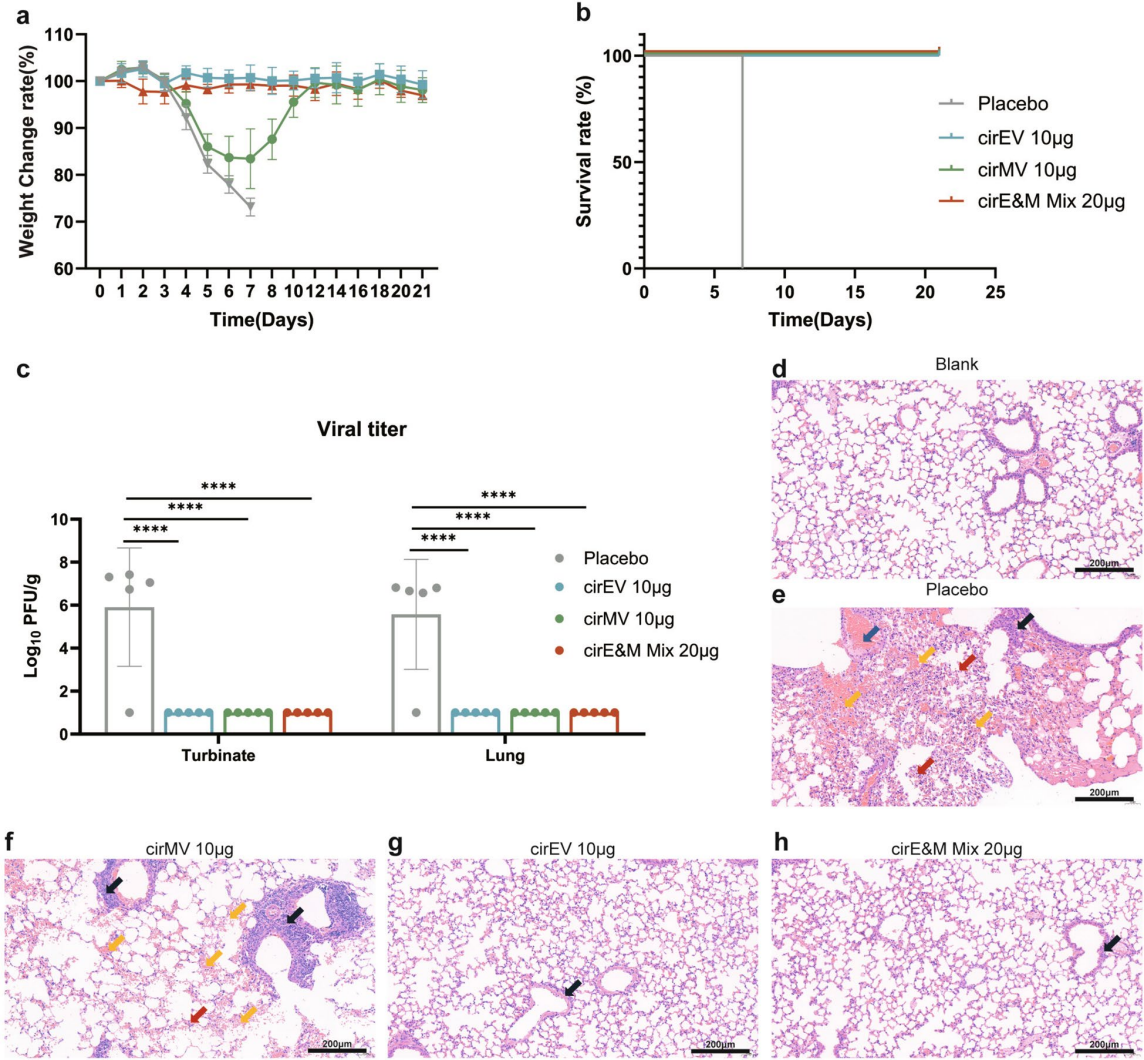
The long-term protection of mice immunized with MPXV Bi-antigen circRNA vaccines. On day 260 post-primary immunization, mice (n = 5) were intranasally challenged with 1 × 10^6^ of PFU VTT in 50 µl volume. On day 7 post-VTT challenge, five mice per group were euthanized to assess viral loads and pathological damage in lung tissues, the remaining five mice per group were monitored for 21 days to record body weight changes and survival rates after VTT challenge. **a** Body weight changes of immunized mice following VTT challenge. **b** Survival of immunized mice challenged with VTT at 260 days after prime immunization. **c** On day 7 post-VTT challenge, VTT titres in the turbinate and the lung tissues were quantified using plaque assay. **d**-**h** H&E staining of lung sections from mice post VTT challenge. Scale bar = 200 μm. The blue arrow shows pulmonary vein congestion, the black arrow shows lymphocyte infiltration, the red arrow shows necrosis in the alveoli and bronchi and the yellow arrow shows alveolar congestion. The data are presented as the mean ± SEM. Significance was calculated using one-way ANOVA with multiple comparison tests (*p < 0.05, **p < 0.01, ***p < 0.001, **** *p* < 0.0001)

The long-term protection of our vaccines was further evaluated in a lethal VTT challenge model. Body weight monitoring indicated that mice immunized with cirEV and cirE&M Mix showed minimal weight loss after VTT challenge, while those receiving cirMV exhibited a distinct pattern of weight loss followed by recovery (Fig. 6a). Mice in the cirMV group reached peak weight reduction on day 7 post-infection, but recovered to baseline by day 12. Importantly, all vaccinated groups provided complete protection against lethal VTT challenge, whereas 100% mortality was observed in the placebo group by day 6 post-challenge (Fig. 6b).

Viral loads in nasal turbinate and lung tissues were measured at day 7 post-infection, and the results showed that all vaccinated groups achieved complete viral clearance, with infectious viral particles remaining below the detection limit (Fig. 6c). Additionally, H&E staining of lung tissues showed that all vaccinated groups exhibited significantly attenuated pulmonary pathology compared to the placebo group, which developed severe pulmonary hemorrhage, tissue necrosis and extensive inflammatory infiltration (Fig. 6d-h). The cirMV group displayed minor histopathological changes, which developed less pulmonary hemorrhage, tissue necrosis and inflammatory infiltration (Fig. 6f). However, cirEV and cirE&M Mix groups preserved near-intact alveolar structures with only scant inflammatory cells (Fig. 6g-h), suggesting an effective long-term protection.

### Safety evaluation of bi-antigen MPXV circular RNA vaccines

**Fig. 7:**
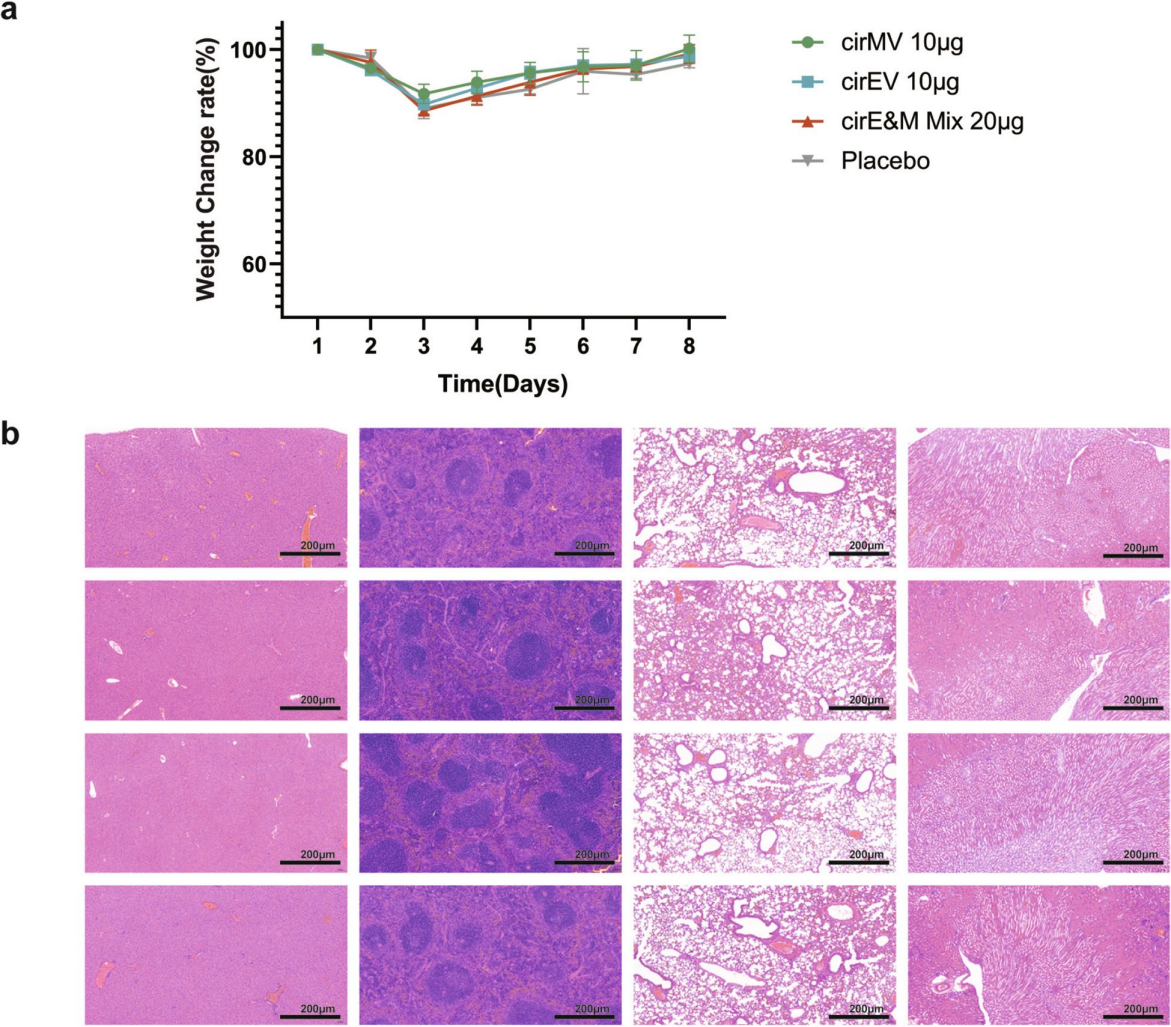
Safety evaluation of Bi-antigen MPXV circular RNA vaccines. **a** Body weight changes of mice following Bi-antigen MPXV circular RNA vaccines inoculation. **b** H&E staining of liver, spleen, lung and kidney sections from mice post inoculation. Scale bar = 200 μm

The safety profiles of the candidate MPXV circular RNA vaccines were further evaluated in immunocompetent mice through comprehensive monitoring of clinical signs, body weight changes, and histopathological examinations. Compared to the placebo group, a single immunization with 10 μg bi-antigen MPXV circRNA vaccines (cirEV & cirMV) or 20 μg quadrivalent vaccine cirE&M Mix did not result in significant weight loss (Fig. 7a), suggesting a low toxicity at the high dose of vaccines. In addition, all mice, including those in the placebo group, showed weight recovery by the third day post-immunization and returned to baseline weight by the seventh day (Fig. 7a). On day 8 post-immunization, liver, spleen, lung, and kidney tissues were collected from all mice and subjected to H&E staining. No significant pathological alterations in any vaccinated groups relative to placebo controls, suggesting those MPXV circular RNA vaccines are relatively safe and caused no tissue damage to mouse (Fig. 7b).

## Discussion

We previously constructed four circRNA vaccines respectively expressing the MPXV proteins A29L, A35R, B6R and M1R and evaluated their protective efficacy in mouse models. The four circRNA MPXV vaccines could induce MPXV antigen-specific humoral and cellular immune responses, resulting in effective protection against VACV in mice. Strikingly, the combination of the four circular RNA vaccines demonstrated superior protection against lethal VACV challenge among all the tested vaccines [22]. To further improve MPXV circRNA vaccine efficacy and reduce production costs, we developed two MPXV vaccines-cirEV and cirMV- expressing MPXV A29L-M1R (IMV antigens) and A35R-B6R (EEV antigens), respectively. The two vaccines alone or in combination can induce strong MPXV antigen-specific humoral and cellular immune responses and provide potential protection against lethal VACV challenge in a dose-dependent manner, and the protective effects of cirEV and cirE&M Mix is superior to that of cirMV. Furthermore, a single 10 μg dose of either cirEV, cirMV, or cirE&M Mix vaccine conferred 100% protection against lethal vaccinia virus challenge at 260 days post-immunization, demonstrating the long-term protection of these multivalent circRNA vaccines for mpox prevention in a VACV model.

Compared to traditional linear mRNA, circRNA exhibits enhanced stability and immune activation potential, making it a highly promising vaccine platform for infectious disease prevention and tumor therapy [18]. In recent years, several circRNA-based vaccines targeting SARS-CoV-2 [24], Zika virus [25] and influenza virus [26] have been developed, demonstrating the ability to elicit both humoral and cellular immune responses, that provide robust protection against virus challenge. CircRNA vaccines also demonstrate prolonged antigen expression, potentially conferring durable protective immunity. Notably, even at lower doses, the circRNA vaccine can induce detectable immune responses [24]. In this study, we first evaluated the antibody and cellular immune responses induced by different dose of cirEV, cirMV and cirE&M Mix in mice. Interestingly, all vaccinated groups elicited robust MPXV A29L- and A35R-specific binding antibody titers, even the minimally dose of the bivalent vaccines (2 μg of each) or cirE&M Mix (4 μg) could induce significantly higher antibody levels compared to the LNP control group. However, these vaccines induced relatively lower antibody levels against M1R and B6R, which may correlate with the low neutralizing antibody titers detected, as M1R was reported to induce a strong humoral immune response [21, 22]. In the bi-antigen circRNA construct, M1R was expressed in tandem with A29L, and unequal translation efficiency or structural constraints associated with polycistronic antigen design may have resulted in suboptimal M1R expression. Besides, improper folding or altered epitope accessibility of M1R following tandem expression may be another reason leading the low titer of neutralizing antibodies.

The cellular immune responses were also detected by ELISpot and intracellular cytokine staining (ICS), and the results demonstrated that all four antigens in each vaccine candidate could induce robust cellular immune responses. Notably, the high-dose formulations (10 μg cirEV or cirMV and 20 μg cirE&M Mix) exhibited superior levels of cellular immunity compared to lower-dose groups. Following lethal VACV challenge, all immunized mice (with the exception of the 2 μg cirMV group) achieved complete protection with rapid weight recovery, and demonstrated total virus clearance in turbinate and lung tissues. The inflammatory responses in lung of high dose of cirMV and cirE&M Mix groups were also significantly attenuated. Remarkably, the 2 μg cirEV formulation conferred complete protection (100%) against lethal VACV challenge, while 2 μg cirMV provided 75% protection, demonstrating the ability of circRNA vaccines to elicit potent immunity even at low doses. However, only the high-dose groups (10 μg cirMV and 20 μg cirE&M Mix) developed low-titer VTT cross neutralizing antibodies, while no MPXV-specific neutralizing antibodies were detected in any vaccinated group. Despite this, complete protection was achieved, cellular immunity likely plays a major role. As we described in our previous study, the neutralizing antibodies produced in response to M1R primarily function to alleviate early symptoms and delay their onset. In contrast, the A35R-dominated cellular immunity serves as the principal mechanism for viral clearance [22]. Although the vaccinated mice had low levels of neutralizing antibodies, the pathological damage in lung tissue was significantly reduced after viral challenge, which also highlights the key role of T cell immunity. In addition, circRNA vaccine can elicit robust antigen-specific T cell responses, which may also mediate viral clearance [22, 27]. Thus, it is necessary to monitor the persistence of MPXV specific T-cell immunity following vaccination [28].

The inherent stability of circRNA-based vaccines enables prolonged antigen expression over time, thereby may facilitating the formation of long-lasting immune memory [16, 29]. Here, we also evaluated the long-term protective efficacy of 10 μg cirEV or cirMV and 20 μg cirE&M Mix at day 260 post first immunization. The MPXV antigen specific binding antibody levels remained relatively high in all groups, but only 10 μg cirMV and 20 μg cirE&M Mix groups showed detectable cross-neutralizing antibodies against VTT, but with no MPXV specific neutralizing antibodies were detected in any of the three immunized groups. However, all three vaccines conferred complete protection and achieved full viral clearance in both nasal turbinate and lung tissues after lethal VACV infection. Moreover, we found that, among the three candidate vaccines, cirEV and cirE&M Mix demonstrated significantly better long-term protective efficacy compared to the cirMV immunization group. The cirMV-immunized mice exhibited the most pronounced weight loss after the lethal-dose VACV challenge, along with slower recovery of body weight and more severe pulmonary inflammation. These results are consistent with our observations from the protection efficacy assessment conducted 2 weeks post the second immunization upon viral challenge. The observed differences could be attributed to functional divergence among the vaccine-encoded antigens. cirMV encodes two MPXV IMV antigens A29L and M1R, while cirEV contains two EEV antigens A35R and B6R. A29L, M1R and B6R and their vaccinia virus homologs A27L, L1R and B5R are reported to contain critical neutralization epitopes thus elicit neutralizing antibodies production [19, 30]. Our preliminary studies found that A35R mainly elicits a potent cellular immune response which is responsible for the clearing the virus from infected tissues [22]. In the long-term immune protection evaluation, the humoral immune responses did not show significant differences among the three groups, suggesting that the observed protection may be mediated by vaccine-induced cellular immunity. Previous studies have shown that cellular immune responses to traditional vaccines are long-lived and can persist for decades after immunization [31, 32]. Thus, the dynamics of cellular immune responses induced by those circRNA MPXV multivalent vaccines should be monitored in subsequent studies.

One important limitation of this study is that protective efficacy was evaluated using a lethal VACV challenge model rather than authentic MPXV infection. Although VACV is commonly used as a surrogate orthopoxvirus model, protection against VACV does not fully recapitulate MPXV infection due to differences in viral tropism and host-virus interactions. Moreover, standard laboratory mice are not naturally susceptible to MPXV, and MPXV challenge studies require specialized animal models and high-level biosafety facilities. Therefore, the conclusions of this study are limited to demonstrating cross-protective efficacy in a VACV model. Future studies using authentic MPXV challenge models will be necessary to validate the translational relevance of these circRNA-based vaccines. Another limitation is that a direct in vivo comparison with nucleoside-modified linear mRNA vaccines was not performed in this study. Previous studies have reported that circRNA vaccines can exhibit enhanced stability and prolonged antigen expression compared with linear mRNA, leading to improved or sustained immune responses in comparable vaccine models [24, 25]. In line with these reports, the robust cellular immunity and long-term protective efficacy observed here support the potential advantages of the circRNA platform. Head-to-head in vivo comparisons between circRNA and linear mRNA vaccines should be performed in our futher work.

In summary, the MPXV bi-antigen circRNA vaccines, especially cirEV and the quadrivalent vaccine cirE&M Mix, can effectively induce robust and durable antigen-specific antibody responses and cellular immune responses and provide full protection against lethal VTT challenge, coupled with viral clearance in tissues. This protective immunity is assumed to be tightly mediated by vaccine-induced immune responses, especially may be associated with vaccine-induced cellular immunity. Importantly, the combination of the two-antigen vaccines (cirE&M Mix) induced equivalent protective efficiency compared to the combination of the four individual circular RNA vaccines(cirMix) as we previously reported [22], which may be superior in cost and manufacture in practical applications. Our results propose circRNA as a promising platform for a safe and effective MPXV even *Orthopoxvirus* vaccine. Future work will assess the protective effects of these vaccines in an MPXV model, providing data to inform future clinical trials.

## Materials and methods

### Cells and viruses

Vero E6 and HEK293T cell lines were cultured in DMEM (Gibco) supplemented with 10% fetal bovine serum (BI) and antibiotics (100 U/mL penicillin and 100 μg/mL streptomycin; BBI) at 37 °C with 5% CO_2_. The VACV Tian Tan (VTT) strain was propagated and titrated in Vero E6 cells. MPXV strain (CSTR:16533.06. IVCAS6.9141), originally isolated from a clinical specimen obtained from a patient in Wuhan, China, was grown in Vero E6 cells for neutralization assays.

### Synthesis and purification of MPXV circRNAs

The EEV-associated antigens (A35R and B6R) and IMV-associated antigens (A29L and M1R) were selected as immunogens, and the antigen-encoding sequences for A35R-B6R and A29L-M1R were cloned into circular RNA vectors via XbaI-mediated plasmid linearization (Thermo FD0685). A35R-B6R and A29L-M1R were constructed as two fusion proteins, with each antigen pair connected by a P2A self-cleaving peptide. In vitro transcription was performed using T7 RNA polymerase (Thermo AMB1335-5) with standard nucleotide triphosphates, followed by DNase I digestion of template plasmids. Purification involved sequential chromatography and enzymatic treatment. First, size exclusion chromatography (Sepax 215,950–30030) at 10 mL/min flow rate removed concatemers and residual fragments using a 75 mM phosphate buffer (pH 7.4). Subsequent affinity chromatography (HiTap NHS-activated HP columns; Cytiva 17,071,601) under lithium chloride buffer (15 mM LiCl, 10 mM Tris, 0.5 mM EDTA) eliminated precursor RNAs. Post-purification, RNase R (37 °C, 30 min) was optionally applied to remove nicked RNA, followed by concentration adjustment with Zymo R1019 columns.

### LNP encapsulation

CircRNA was encapsulated into lipid nanoparticles (LNPs) using the NanoAssemblr Ignite platform as previously described [33, 34]. Briefly, circRNA in acidic buffer (pH 4.0) was mixed with an ethanol-based lipid solution (ALC-0315:DSPC:Cholesterol:ALC-0519, molar ratio 46.3:9.4:42.7:1.6) via microfluidic mixing. LNPs were dialyzed against 50 mM Tris–HCl (pH 7.0) and cryopreserved at −80 °C. Encapsulation efficiency and hydrodynamic diameter were analyzed using a Stunner™ system.

### CircRNA-LNP transfection analysis

At 70–80% confluency, HEK293T cells were transfected with 2.5 μg circRNA per well (6-well plate) using Lipofectamine 3000 (Thermo). Forty-eight hours post-transfection, Immunofluorescence (IFA) was used to detect the antigen expression of each vaccine candidate. In brief, circRNAs transfected cells were fixed with 4% paraformaldehyde in PBS for 30 min at room temperature, followed by permeabilization with 0.5% Triton X-100/5% BSA for 1 h at 37 °C. After blocking with 5% BSA for 1 h, samples were incubated with MPXV-specific polyclonal antibodies for 1 h at 37 °C. Secondary staining was performed using Alexa Fluor 488-conjugated anti-rabbit IgG (Abcam), with nuclear counterstaining by DAPI prior to fluorescence imaging (EVOS FL Auto 2.0, Thermo Fisher).

### Mouse vaccination

Six-week-old female BALB/c mice were randomly allocated into experimental cohorts. The bivalent vaccine groups received intramuscular injections of either cirEV or cirMV at doses of 2 μg or 10 μg, respectively. The quadrivalent group (cirE&M Mix) received three escalating doses: 4 μg (2 + 2 μg), 10 μg (5 + 5 μg), or 20 μg (10 + 10 μg). The control group received empty lipid nanoparticles (LNPs) matched in total mass. A prime-boost immunization regimen was implemented at a 2-week interval. Serum samples and spleen were collected at day 28 post first immunization and subjected to antibody or cellular immune responses detection (Table.S1).

For long-term immunization evaluation, mice were bled at day 260 post-primary immunization to assess MPXV specific antibody responses, followed by a lethal VTT challenge to evaluate protective efficacy (Table.S2).

### Enzyme-linked immunosorbent assay (ELISA)

ELISA assay was used to detect MPXV antigen-specific binding antibodies levels in mouse serum. The 96-well ELISA plates were coated with recombinant antigens (1 μg/mL) overnight at 4 °C. After washing with PBST, the plates were blocked with 1% BSA for 1 h at 37 °C. Mouse sera were serially diluted fourfold (starting from 1:100) and incubated at 37 °C for 1 h. MPXV antigen-specific antibodies were detected using HRP-conjugated goat anti-mouse IgG (ABclonal; 1:10,000) followed by TMB substrate (Solarbio). Absorbance was measured at 450/630 nm using an ELISA plate reader (BioTek SYNERGY h1). Endpoint titers were defined as the highest serum dilution exceeding 2.1 × negative control values.

### Live-virus neutralization assay

Neutralizing antibody titers were evaluated using plaque reduction neutralization tests (PRNT) as previously reported [22]. Briefly, Vero E6 monolayers were infected with twofold serially diluted serum-virus complexes (200 PFU/well VTT or 70 PFU/well MPXV; 1 h, 37 °C). Following adsorption, 1% methylcellulose overlay was added for plaque formation (48 h). After fixation (10% formalin) and crystal violet staining, PRNT_50_ titers were calculated.

### ELISPOT assay

Isolated splenocytes (2.5 × 10^5^ cells/well) were stimulated with peptide pool for the MPXV-A29L, A35R, M1R, and B6R protein (2 μg/mL; DGpeptides) and assessed using precoated ELISPOT kits according to the manufacturer’s protocol. Following overnight incubation at 37℃, spots were developed with AEC substrate and quantified using an AID ELISPOT reader.

### Flow cytometry

Splenocytes (1 × 10^7^/well) were stimulated for 6 h with MPXV peptides (MPXV-A29L, A35R, M1R, and B6R, 2 μg/mL) and stained with viability dye FVS700 (BD). After Fc receptors blocking, cells were surface-stained with anti-CD3-FITC, CD8-PerCP-Cy5.5, and CD4-BV421 (BD Pharmingen) followed by intracellular staining for cytokines (PE-IL-2, APC-TNF-α, BV650-IL-4, BV786-IFN-γ, BD Pharmingen). Data were acquired using a BD FACSCanto II flow cytometer.

### Mouse challenge

BALB/c mice (n = 6) receiving MPXV circRNA vaccines immunization were challenged via intranasal inoculation with 1 × 10^6^ PFU VTT under anesthesia at day 30 post-first vaccination. Body weight was monitored daily until day 28 post-challenge. Subgroups were euthanized at day 7 for tissue collection (nasal turbinate and lungs) for viral load measurement. Lung sections were processed for H&E staining.

### Mouse challenge for long-term vaccination

BALB/c mice (n = 10) receiving MPXV circRNA vaccines immunization were challenged via intranasal inoculation with 1 × 10^6^ PFU of VTT under anesthesia at day 260 post-first vaccination. Body weight was monitored daily until day 21 post-challenge. Subgroups were euthanized on day 7 for tissue collection (nasal turbinates and lungs) for viral load measurement. Lung sections were processed for H&E staining.

### Viral load determination

Viral DNA was extracted (Vazyme kits) and quantified via qPCR using M1R-specific probes (Table.S3). Infectious viral titers were determined by plaque assay and expressed as PFU per gram of tissue.

### Histological analysis

Lung tissues were fixed in 4% paraformaldehyde, embedded in paraffin, and sectioned at 5 μm thickness for Hematoxylin and Eosin (H&E) staining and histopathological evaluation.

### In vivo toxicity evaluation

To evaluate in vivo toxicity of those circRNA vaccines, BALB/c mice (n = 3) were intramuscularly immunized with either 10 μg of cirEV, 10 μg of cirMV, or 20 μg of cirE&M Mix. Body weight was monitored daily post-immunization. On day 7 post-immunization, mice were euthanized to collect serum for biochemical analysis of key metabolic markers. Concurrently, major organs (liver, spleen, lungs, and kidneys) were harvested for histopathological assessment using H&E staining.

### Statistical analysis

Statistical analyses were performed using GraphPad Prism 9.0 (GraphPad Software). Data are presented as mean ± SEM. Differences between groups were assessed by one-way ANOVA with statistical significance defined as *p* < 0.05.

## Supplementary Information

Supplementary Material 1.

## Data Availability

All the data from the corresponding authors are available upon reasonable request.
