# Supplementary material for: CircRNA based bi-antigen vaccines against mpox virus induce potent and durable cross-protection in mice
Source: Mol Biomed. 2026 May 28;7:79. doi: 10.1186/s43556-026-00474-9 (PMC13219560; doi:10.1186/s43556-026-00474-9)
Supplement: Supplementary file 1 — Supplementary Material 1. [file 43556_2026_474_MOESM1_ESM.docx]

**Table S1** **Group of mice for immunization with different doses**

| Group Number | Vaccine Components | Antigens | Dose |
| --- | --- | --- | --- |
| 1 | Placebo | N/A | Equal as 20μg |
| 2 | cirEV | A35R+B6R | 2 μg |
| 3 |  |  | 10 μg |
| 4 | cirMV | A29L+M1R | 2 μg |
| 5 |  |  | 10 μg |
| 6 | cirE&M Mix | A35R+B6R& A29L+M1R | 2 μg |
| 7 |  |  | 10 μg |
| 8 |  |  | 20 μg |

**Table S2** **Group of mice for long-term immunization**

| Group Number | Vaccine Components | Antigens | Dose |
| --- | --- | --- | --- |
| 1 | Placebo | N/A | Equal as 20μg |
| 2 | cirEV | A35R+B6R | 10 μg |
| 3 | cirMV | A29L+M1R | 10 μg |
| 4 | cirE&M Mix | A35R+B6R& A29L+M1R | 20 μg |

**Table.S3 Primers for qPCR**

| Primer | Sequence |
| --- | --- |
| L1R-F | AGTGGATTAACACCGGAACAA |
| L1R-R | CGACCGCGCTAGAATTACAA |
| L1R-probe | CGTACCAGCTATGTTTACTGCTGCGT |

**
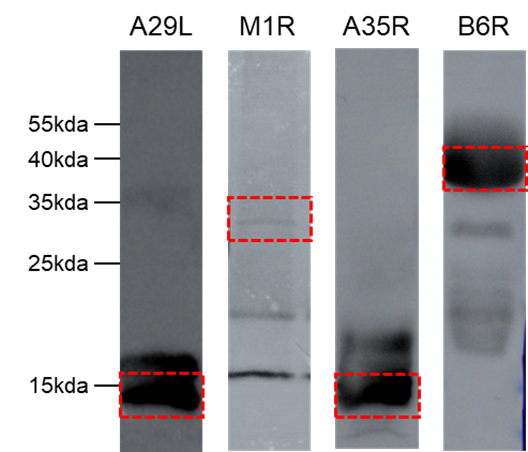
**

**Figure. S1 Characterization of MPXV circRNA vaccines.** The expression of the MPXV-specific antigens A29L, M1R(cirMV), A35R, and B6R(cirEV) in HEK293T cells were detected by western blotting.


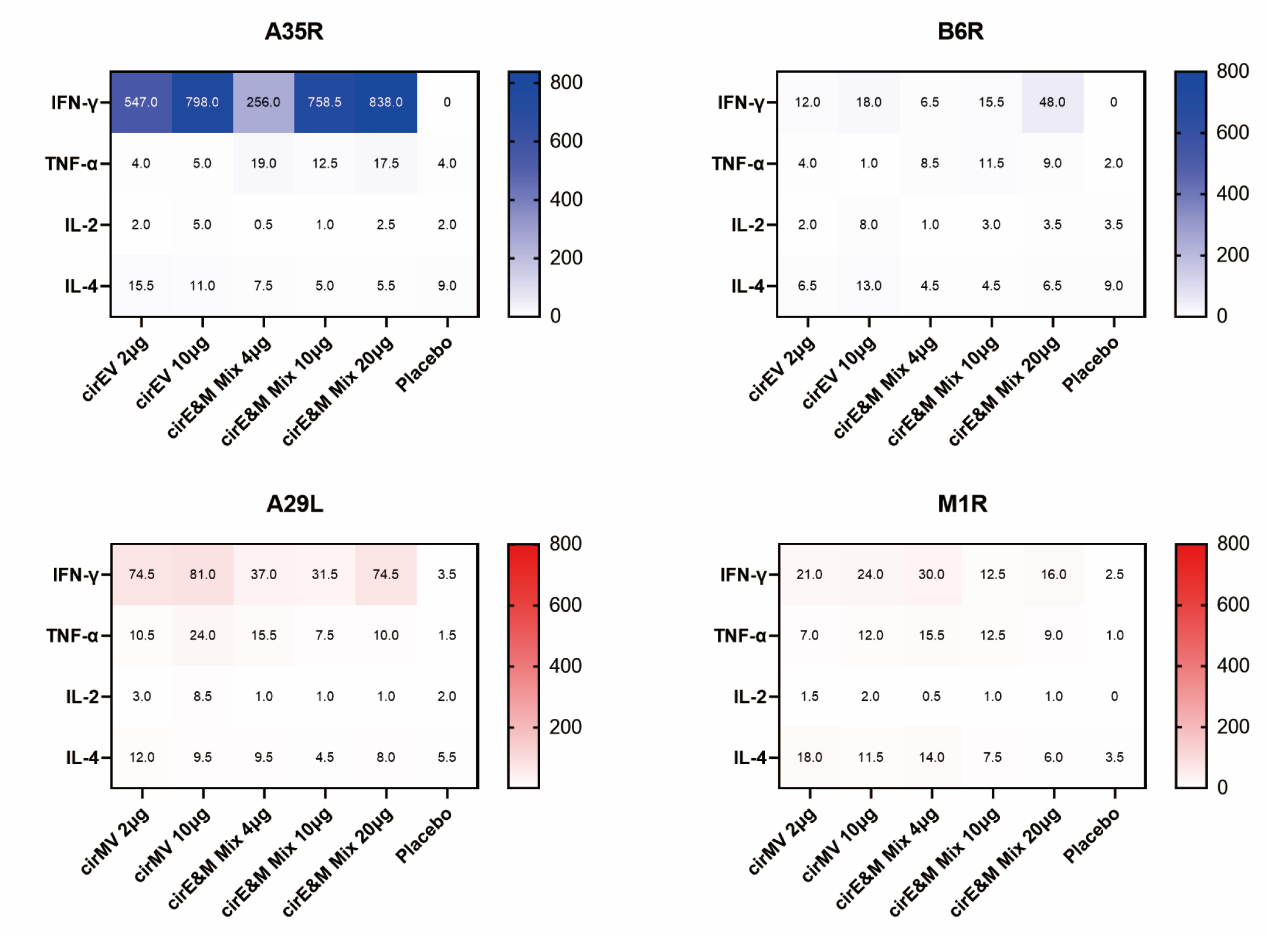


**Figure. S2 Heatmap of cellular immune responses in mice following immunization with bi-antigen MPXV circRNA vaccines as assessed by ELISpot.** Values shown represent the mean number of spots detected per 2.5 × 10⁶ splenocytes. Column titles indicate the antigens used for cell stimulation; blue denotes EEV-derived antigens and red denotes IMV-derived antigens. Darker color intensity corresponds to a higher number of spots.


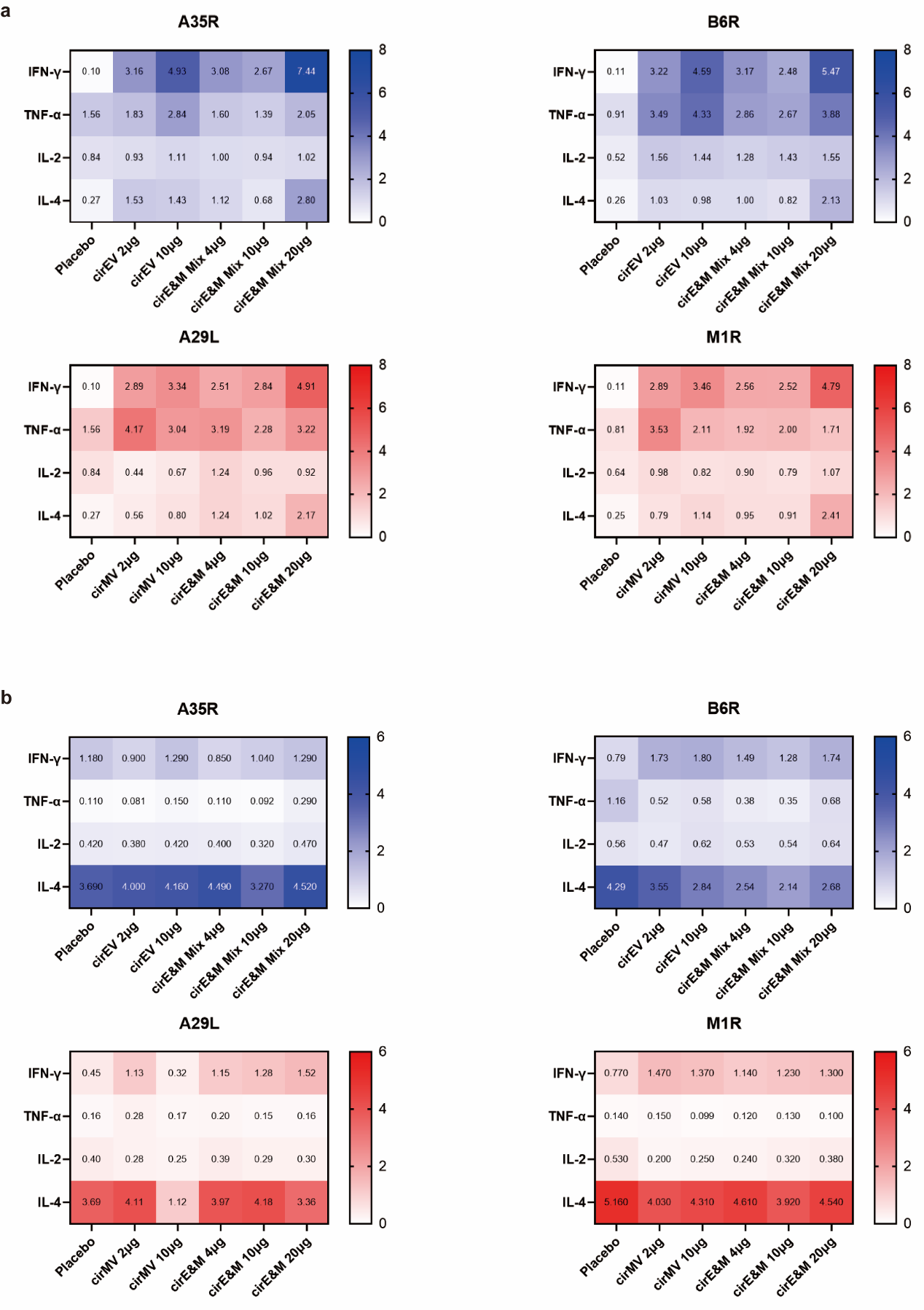


**Figure. S3 Heatmap of cellular immune responses in mice following immunization with bi-antigen MPXV circRNA vaccines as measured by intracellular cytokine staining (ICS).** Values shown represent the mean percentage of positive cells detected by ICS. Column titles indicate the antigens used for cell stimulation; blue denotes EEV-derived antigens and red denotes IMV-derived antigens. Darker color intensity corresponds to a higher proportion. (a) heat map of CD8^+^ cells. (b) heat map of CD4^+^ cells.
